# Integrated Microbiome-Metabolomics Analysis Reveals the Potential Mechanism of Dandelion Root Polysaccharides to Ameliorate Ulcerative Colitis

**DOI:** 10.3390/metabo14070351

**Published:** 2024-06-22

**Authors:** Shengkun Yan, Rong Dong

**Affiliations:** Agricultural Mechanization Institute, Xinjiang Academy of Agricultural Sciences, Urumqi 830091, China

**Keywords:** dandelion root polysaccharides, ulcerative colitis, intestinal flora, metabolomics, correlation

## Abstract

In the conducted research, a murine model for ulcerative colitis (UC) was established utilizing dextran sodium sulfate (DSS) to investigate the therapeutic potential of dandelion root polysaccharide extracts on this disease. This study employed an analysis of gut microbiota composition and serum metabolomics to understand the biochemical effects of these polysaccharides. Sequencing of the 16S ribosomal DNA component indicated an increased presence of Bacteroides in the DSS-treated model group, contrasting with a significant enhancement in Faecalibaculum populations in mice treated with dandelion root polysaccharides (DPs). This shift suggests a pivotal role of DPs in elevating fecal N-butyric acid levels—a crucial factor in the maintenance of gut microbiota equilibrium. Through metabolomic profiling of serum, this research identified distinct metabolic changes across the control, DSS model, and DP treatment groups, highlighting four major differential metabolites: (2S)-2-amino-3-[[(2R)-2-butanoyloxy-3-propanoyloxypropoxy]-hydroxyphosphoryl]oxypropanoic acid; (1R,8S,9S)-3,4-dihydroxy-8-methoxy-11,11-dimethyl-5-propan-2-yl-16-oxatetracyclo [7.5.2.01,10.02,7]hexadeca-2,4,6-trien-15-one; Aspartylasparagine; and Nap-Phe-OH. These metabolites are implicated in mitigating oxidative stress, suggesting that DPs facilitate a protective mechanism for the intestinal lining through various biochemical pathways. Additionally, a notable correlation was established between the altered gut microbiota and the serum metabolomic profiles, underscoring the intricate interplay between these two biological systems in the context of UC. This study’s outcomes illustrate that UC induces significant alterations in both gut microbiota and metabolic signatures, whereas dandelion root polysaccharides exhibit a profound ameliorative effect on these disruptions. This investigation underscores the therapeutic promise of dandelion root polysaccharides in the management of UC by modulating gut microbiota and metabolic pathways.

## 1. Introduction

Documented in ancient texts such as the *Compendium of Materia Medica*, dandelion has been recognized for its dual utility as both a nutritional and medicinal herb [1]. This plant is celebrated for its comprehensive therapeutic benefits including, but not limited to, anti-cancer, anti-inflammatory, immunomodulatory, and antibacterial properties, in addition to its ability to alleviate swelling and disperse nodules [2]. Dandelion is particularly noted for its rich content of bioactive compounds, with polysaccharides being exceptionally abundant, constituting 30% to 50% of its dry weight [3]. Among its parts, the root stands out for its high polysaccharide concentration. Dandelion polysaccharides may have therapeutic effects on the gastrointestinal tract, and the related mechanisms include reducing the production of pro-inflammatory factors, reducing the expression of reactive proteins, reducing oxidative stress, and regulating intestinal flora [4]. Tian Hua et al. [4] confirmed through mouse experiments that dandelion can directly or indirectly act on the production of gastrointestinal-related inflammatory mediators and inflammatory factors. Natchanok et al. [5] extracted three kinds of dandelion polysaccharides by hot water extraction and ethanol precipitation and tested the immunostimulating activity of polysaccharides on mouse macrophage RAW264.7 through in vitro experiments. Le [6] determined the in vitro antioxidant and in vitro hypoglycemic properties of purified monomer of dandelion leaf polysaccharide and found that dandelion leaf polysaccharide had certain free radical scavenging and α-glucosidase inhibition abilities. Bo [7] has shown that dandelion polysaccharides can remove free radicals and avoid body damage by enhancing the activity of antioxidant enzymes in the body. Yani et al. [8] found that dandelion polysaccharide may reduce intestinal inflammatory response and tissue damage by reducing the secretion of inflammatory cytokines in mice models of microbial dysregulation and thus play a therapeutic role in intestinal dysfunction caused by microbial dysregulation. In summary, dandelion polysaccharide has a certain alleviating effect on gastrointestinal diseases, but there are no studies on the efficacy and application of dandelion polysaccharide in ulcerative colitis.

Ulcerative colitis (UC), a chronic condition characterized by inflammation of the colon, is profoundly influenced by the imbalance of gut microbiota [9]. This imbalance disrupts the symbiotic equilibrium between the intestinal microbiota and the host, essential for maintaining intestinal barrier integrity, modulating immune functions, and facilitating metabolic processes [10]. In UC, the disruption leads to a proliferation of pathogenic bacteria alongside a reduction in beneficial microbes, potentially initiating or exacerbating inflammatory processes [8]. Critical to the maintenance of a healthy gut environment, bacteria such as Bifidobacteria, Eubacterium, and Roseburia are instrumental in the production of short-chain fatty acids, including acetic, propionic, butyric, and isobutyric acids [11,12]. These acids are pivotal for the metabolic health of the host. However, in UC, a marked decrease in these beneficial bacteria results in lowered levels of these important fatty acids, adversely affecting the intestinal cells and prompting inflammatory responses [13]. Additionally, the link between the presence of Clostridium spp. bacteria and increased levels of equine uric acid has been noted as potentially significant in the pathology of UC [14]. The elevation of equine uric acid, produced by the bacterial metabolism of choline, correlates with the severity of UC symptoms [15]. Thus, alterations in the Clostridium spp. population may directly influence UC’s pathological mechanisms by modifying the concentrations of equine uric acid, further affecting the disease’s progression. This nuanced understanding of UC underscores the intricate interactions between gut microbiota and the host’s health, highlighting the critical role of microbial balance in preventing and managing inflammatory bowel diseases. In individuals with UC, the gut microbiota’s role extends to the metabolism of choline, trimethylamine, and methylamine—processes that may become disrupted. Metabolites derived from choline, specifically trimethylamine (TMA) and trimethylamine-N-oxide (TMAO), are implicated in elevating the risk of cardiovascular diseases [16]. This imbalance, known as dysbiosis, in UC patients can lead to alterations in the levels of these metabolites, potentially exacerbating the disease’s progression and impacting cardiovascular wellness. Additionally, the metabolism of tryptophan, an indispensable amino acid, plays a crucial part in UC. Tryptophan’s metabolites, including indoles, are vital for maintaining gut health, offering anti-inflammatory and immunomodulatory benefits [17]. They safeguard the intestinal bacterial membrane’s integrity, diminish the adhesion of harmful bacteria, and enhance the production of anti-inflammatory interleukins while reducing pro-inflammatory factors. In UC sufferers, an imbalance in gut microbiota may impair tryptophan metabolism, resulting in a reduction of these active metabolites. This decrease weakens the gut’s protective mechanisms and intensifies inflammation [18].

Understanding the intricacies of UC surpasses the capacity of individual histological assessments, demanding a more comprehensive approach. The integrated application of multi-omics techniques sheds light on UC complex pathology from various angles, offering a robust framework for investigating its emergence and evolution. Metabolic regulation in the host involves a delicate balance between genetically encoded pathways and those influenced by the microbial genomes within the gut microbiota. This “second genome”, composed of a diverse community of gut microorganisms, plays a pivotal role in the host’s metabolism, influencing a range of physiological responses [19]. Metabolomics, focusing on the fluctuation of endogenous metabolites in response to both genetic and environmental factors, emerges as a key player in decoding these interactions [20]. It has been revealed that alterations in specific serum metabolites, including phenylacetic acid, hydroxyphenylacetic acid, 3-indoleacetic acid, succinic acid, fenugreek acid, and phenylpropionic acid, can be traced back to metabolic activities of both the host and its gut microbiota, particularly pointing to the roles of Clostridium spp. and Mycobacterium spp. in amino acid metabolism [21]. Explorations into natural remedies, such as the use of red dates to address gut flora imbalances in splenic deficiency syndrome, highlight the potential of specific microbial strains like *Aerococcus* spp. to modulate disease-related metabolic pathways [22]. This underscores the utility of combining metabolomics with gut microbiota analysis to not only advance the understanding of natural product efficacy but also to uncover the underlying mechanisms of action. Such an approach, merging insights from metabolomics and microbiome analysis, is yet underexplored in the context of elucidating the effects of polysaccharides on UC, marking a promising avenue for future research. This integrated strategy not only broadens the scope of UC studies but also enriches the methodology for dissecting the multifaceted interactions between host metabolism and gut microbiota dynamics.

In the author’s prior research, DPs significantly enhanced the antioxidant capacity of the cells and the ability to resist iron-induced death in vitro, thus providing a protective barrier for the intestinal epithelium of mice to prevent the typical damage of mice in the UC scenario, suggesting that the intestinal microflora, the metabolic activities of host cells, and their interactions may jointly maintain the intestinal REDOX balance [23]. In this investigation, the employment of a dextran sodium sulfate (DSS)-induced model of UC in mice provided the framework for an in-depth exploration of how dandelion root polysaccharides impact the gastrointestinal microbiome’s diversity and richness. This study meticulously characterized the intestinal microbiota and assessed metabolic profiles by collecting fecal and serum samples, utilizing 16S ribosomal RNA sequencing for comprehensive microbial analysis alongside ultra-performance liquid chromatography coupled with quadrupole time-of-flight mass spectrometry (UPLC-Q-TOF-MS/MS) for precise metabolite quantification. This research aimed to pinpoint the specific microbial shifts induced by dandelion root polysaccharide intervention and to delineate its regulatory influences on the gut microbiome in a UC context. Furthermore, the study ventured to examine the protective attributes of dandelion root polysaccharides against UC by analyzing alterations in metabolite concentrations and metabolic pathways, establishing correlations between predominant microbial species and key metabolic changes. This approach sought to uncover the mechanisms through which dandelion root polysaccharides might mitigate the effects of UC in mice, offering insights into potential therapeutic applications.

## 2. Materials and Methods

### 2.1. Preparation of Dandelion Root Polysaccharides

Dandelion root, acquired from Hotan, Xinjiang, China, in July 2022, was processed to extract dandelion polysaccharides (DPs) using an established method from prior research [23]. The process began with a thorough rinse of the roots in distilled water, followed by drying at 70 °C. Once dry, the roots were pulverized into a powder. This powder then underwent extraction and filtration using 95% ethanol, separating out the precipitate. The filtered solid residues were air-dried, then re-extracted with distilled water. The extract was centrifuged at 4000 RPM for 15 min, with the procedure repeated twice to ensure thoroughness. The supernatant’s volume was condensed to about one-tenth its original size, and solids were removed via centrifugation under the same conditions. Ethanol, four times the volume of the supernatant, was added to induce precipitation. The resulting precipitate was sequentially washed with petroleum ether, acetone, and pure ethanol, then freeze-dried to yield the crude polysaccharide, referred to as DP.

### 2.2. Animal Experiment Design

A total of twenty-four male mice of the C57BL/6 strain, all six weeks of age and with a body mass ranging from 18 to 22 g, were procured from Chengdu Dossy Experimental Animals Co., Ltd. (Chengdu, China), which holds a certification SCXK (Szechwan)2020-030. These animals were accommodated in a facility that maintains a specific pathogen-free (SPF) status, under a regulated cycle of 12 h of light followed by 12 h of darkness. The experimental protocols employed in this study were in strict compliance with the guidelines for animal care issued by the Health Ministry of China. Approval for these experiments was granted by the animal ethics committee of Huaxi Hospital at Sichuan University, documented under approval code 20230817003.

**Figure 1:**
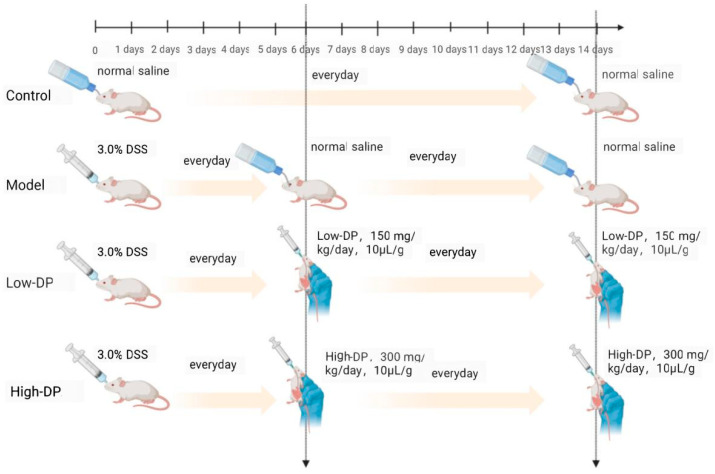
Schematic diagram of the treatment schedule for UC. Drawing created using BioRender.com.

As shown in Figure 1, following a seven-day period for adaptation, mice were allocated randomly into four groups with six animals per group: a Control group, a Model group with DSS-induced UC, and two groups with DSS-induced UC receiving 150 mg/kg/day and 300 mg/kg/day of dandelion polysaccharides, labeled as Low-DP dose and High-DP dose. The Control group were administered 0.2 mL of normal saline per mouse exclusively throughout the experiment. In contrast, for the initial five days, the Model group and both DP dose groups were given a 3.0% DSS solution via oral administration one time every day. From the sixth to the fourteenth day, animals in the DP dose groups were administered DSS, at concentrations determined by their group assignment: either 150 mg/kg/day or 300 mg/kg/day. Upon concluding the experiment, euthanasia was carried out by administering an injection of 1% sodium pentobarbital at a dose of 50 mg/kg, followed by inhalation of carbon dioxide. Subsequently, collection of tissue specimens, fecal samples, and blood was performed. Detailed recordings were maintained throughout the duration of this study for each subject’s body weight, disease activity index (DAI)—which encompasses weight variation, fecal consistency, and presence or absence of blood in the feces—as well as the length of the colon.

### 2.3. Collection of Blood and Feces Samples

Prior to euthanasia, the animals underwent a 24-h feeding period, followed by an overnight fasting period without access to water. Subsequently, feces were directly collected from the intestinal tract of the mice and immediately placed into sterile 2 mL centrifuge tubes, then stored at −80 °C for future analysis. The mice were then euthanized by inhaling carbon dioxide. Blood was extracted from the mice orbital sockets and centrifugated at a speed of 2500 rpm for 30 min at ambient temperature. The obtained serum was then stored at −80 °C for future analysis.

Mouse feces were collected directly from the intestinal tract and subsequently placed into sterile 2 mL centrifuge tubes, which were then stored at −80 °C for future analysis. Following a 24-h feeding period, mice underwent a fasting period overnight without access to water. 

### 2.4. Fecal Bacteria 16S rRNA Sequencing Analysis

From each group, six fecal samples underwent random selection. The genomic DNA was extracted using Tiangen Magnetic Bead Method Fecal Genome DNA Extraction Kit (DP712). The purity and concentration of the extracted DNA were assessed through agarose gel electrophoresis. An appropriate volume of the DNA was then diluted with sterile water to achieve a concentration of 1 ng/μL in a centrifuge tube. This diluted DNA served as the template for polymerase chain reaction (PCR) amplification, employing specific primers that include a barcode. These primers, designed for selected sequencing regions, were 515F (5′-barcode-GTGCCAGCMGCCGCGGTAA-3′) and 806R (5′-GGACTACHVGGGTWTCTAAT-barcode-3′). The PCR amplification used the Phusion^®^ High-Fidelity PCR Master Mix with GC Buffer from New England Biolabs, ensuring high efficiency and fidelity. Electrophoresis on a 2% agarose gel was used to confirm the PCR products, which were then purified using magnetic beads. Quantification followed purification, performed with enzyme labeling. Based on PCR product concentrations, aliquots were prepared, thoroughly mixed, and their integrity was confirmed again by 2% agarose gel electrophoresis. Target bands were then extracted using a gel recovery kit from Qiagen. For library construction, the TruSeq^®^ DNA PCR-Free Sample Preparation Kit was employed. Libraries were quantified using Qubit and Q-PCR. Upon meeting quality standards, sequencing was conducted on a NovaSeq6000 platform. Prior to sequencing, all PCR products underwent standardization and purification.

Following the separation of data derived from downlinked sequences utilizing barcode and PCR amplification primer sequences, each sample’s data underwent truncation of these identifying sequences. Subsequently, FLASH (v1.2.11, http://ccb.jhu.edu/software/FLASH/ (accessed on 13 May 2024) facilitated the splicing of reads for each sample, generating the initial set of sequences, termed as Raw Tags. These Raw Tags, post-splicing, underwent a series of quality control measures including sequencing data verification, filtration, cropping, further splicing, and chimera removal as per the protocols established by Qiime (V1.9.1, http://qiime.org/scripts/split_libraries_fastq.html (accessed on 13 May 2024), resulting in a refined dataset of sequences known as Clean Tags. The clustering of Effective Tags from all samples was executed using the Uparse algorithm within USEARCH v7 (http://www.drive5.com/usearch/ (accessed on 13 May 2024), selecting the most frequently occurring sequence within OTUs as the representative sequence, adhering to the algorithm’s core principles. For species annotation, OTU/ASV sequences were analyzed using the Mothur approach against the SSUrRNA database from SILVA138.1 (http://www.arb-silva.de/ (accessed on 13 May 2024) with a specificity range set between 0.8 and 1, facilitating the acquisition of detailed taxonomic information at each classification level. Subsequent steps involved employing MAFFT (v7.490, https://mafft.cbrc.jp/alignment/software/ (accessed on 13 May 2024) for efficient alignment of all representative OTU/ASV sequences, thereby aiding in the systematic analysis of the sequences. Analysis of both α diversity (encompassing observed_otus, Shannon, Simpson, and Chao 1 metrics) and β diversity (through the weighted UniFrac distance matrix) was conducted at the OTU level, with results visualized via principal coordinate analysis. The creation of PCA and PCoA diagrams was accomplished using R software (Version 4.1.2). Additionally, LEfSe utilized linear discriminant analysis (LDA) for identifying taxa that significantly differentiate between sample groups.

### 2.5. Extraction and Detection of Serum Metabolites

The serum specimens were defrosted on ice until completely devoid of ice crystals. Post-thaw, the specimen was agitated for 10 s using a vortex to ensure thorough mixing. Subsequently, 50 µL of this specimen was transferred into a pre-numbered centrifuge tube. A total of 300 µL of a 20% acetonitrile methanol solution was introduced into the tube, serving as the internal standard because of its efficacy in enhancing metabolite extraction efficiency and providing a stable reference signal across a broad range of metabolites for accurate quantification in LC-MS analysis. The mixture was agitated for 3 min, followed by centrifugation at 4 °C at a speed of 12,000 rpm for 10 min. A total of 200 µL of the clear supernatant was carefully transferred to another centrifuge tube bearing the same number and refrigerated at −20 °C for 30 min. This tube was further centrifuged at 4 °C at 12,000 rpm for 3 min, then 180 µL of the resulting supernatant was moved via pipette into the designated sample vial’s inner liner for LC-MS subsequent instrumental analysis. For quality control (QC), 30 μL of the supernatant from each sample was mixed to form a sample pool for monitoring the stability of sequence analysis. All the above operations were conducted on ice. LC-MS system analytical conditions were as follows.

UPLC: column, Waters ACQUITY UPLC BEH C18 1.8 µm, 2.1 mm × 100 mm; column temperature, 40 °C; flow rate, 0.4 mL/min; injection volume, 2 µL; solvent system, water (0.1% formic acid): acetonitrile (0.1% formic acid). The column was eluted with 5% mobile phase B (0.1% formic acid in acetonitrile) at 0 min followed by a linear gradient to 90% mobile phase B (0.1% formic acid in acetonitrile) over 11 min, held for 1 min, and then back to 5% mobile phase B within 0.1 min, held for 1.9 min, then rapidly returned to starting conditions.

MS Conditions (AB): The data acquisition was conducted using the information-dependent acquisition (IDA) mode using Analyst TF 1.7.1 Software (Sciex, Concord, ON, Canada). The source parameters were set as follows: ion source gas 1, 50 psi; ion source gas 2, 50 psi; curtain gas, 35 psi; TEM temperature was 550 °C; declustering potential, 60 V, or −60 V in positive or negative modes, respectively; and ion spray voltage floating, 5000 V or −4000 V in positive or negative modes, respectively. The TOF MS scan parameters were set as follows: mass range, 50–1000 Da; accumulation time, 200 ms; and dynamic background subtract, on. The product ion scan parameters were set as follows: mass range, 25–1000 Da; accumulation time, 40 ms; collision energy, 30 or −30 V in positive or negative modes, respectively; collision energy spread, 15; resolution, UNIT; charge state, 1 to 1; intensity, 100 cps; exclude isotopes within 4 Da; mass tolerance, 50 mDa; and maximum number of candidate ions to monitor per cycle, 12.

Mass spectrometer output was first transformed mininto mzXML format utilizing ProteoWizard. Subsequently, XCMS 4.2.2 software was employed for peak extraction, alignment, and retention time adjustment. Peak areas underwent normalization through the “SVR” technique, and peaks exhibiting over 50% absence in any sample group were excluded. Following these preparatory steps, metabolites were identified by consulting the laboratory’s proprietary databases, supplemented by high resolution self-built library, DB—all public library, AI prediction library, and MetDNA2.0 for comprehensive coverage. Orbitrap Liquid Chromatography-Mass Spectrometry (LC-MS) data analysis was further refined to identify metabolic pathways using both OPLS-DA and KEGG pathway analysis, performed in R software (Version 4.1.2). The Rich Factor within KEGG analysis represents the proportion of significantly altered metabolites within a given pathway relative to the total metabolites annotated for that pathway, serving as an indicator of pathway enrichment. A higher Rich Factor signifies greater enrichment, while *p*-values nearing zero denote statistically significant enrichment. The visual representation in figures features dots sized proportionally to the count of significantly enriched metabolites in each pathway. 

### 2.6. Correlation Analysis between Metabolites and Intestinal Flora

In the analysis of metabolites and intestinal flora using the Chiplot online tool, this study employed the Pearson correlation coefficient to assess linear relationships. The coefficient’s value, ranging from −1 to +1, indicates the direction and strength of the correlation. A coefficient greater than zero signifies a positive relationship, while a value less than zero denotes a negative relationship. The significance of these correlations was evaluated using *p*-values, with values less than 0.05 indicating significant correlations and those below 0.01 signifying highly significant correlations. Differential metabolites were identified with a *p*-value < 0.01, VIP > 1, and a fold change > 4 or <0.25 between control and model groups.

### 2.7. Data Analysis

The majority of the experimental results were processed and visualized with the use of Origin 2021 software, while statistical evaluations were conducted employing one-way ANOVA followed by Duncan’s multiple range test for identifying statistical disparities, using the Chiplot online tool to normalize, analyze, and illustrate data related to the gut microbiota. The presentation of all data adhered to the format of mean ± SEM. Statistical significance was determined based on *p*-values, with *p* < 0.05 indicating a significant discrepancy and *p* < 0.01 denoting a highly significant discrepancy. The notations * *p*, ** *p* < 0.01, and *** *p* were used to signify the levels of significance in comparison to the Model group.

## 3. Results and Discussion

### 3.1. The Effects of DPs on α Diversity of Intestinal Flora in Mice

**Figure 2:**
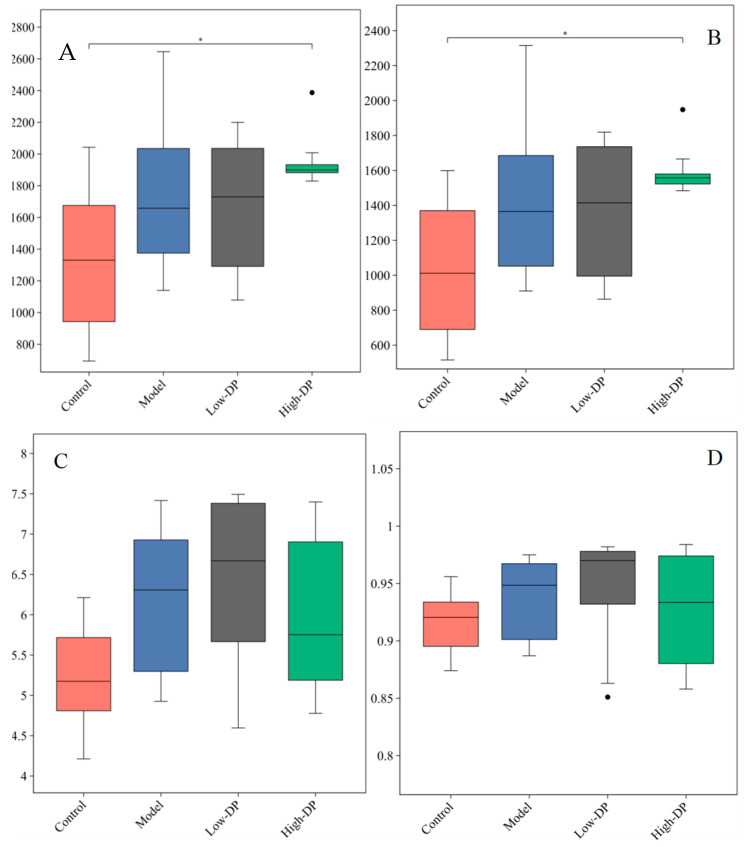
Alpha diversity of gut microbiota (*n* = 6). (**A**) Chao1 index; (**B**) Observed _otus index; (**C**) Shannon index; (**D**) Simpson index. ”*” Indicates significance between the two groups (*p* < 0.05).

Alpha diversity metrics serve as tools to explore the variety and abundance of microbial entities within a given ecosystem or locale by assessing the diversity within a singular sample. These metrics, namely the observed_otus and Chao1 indices, shed light on the species richness present in each sample, offering insights into both the observed and potential species count. The observed_otus index specifically quantifies the species directly detected, whereas the Chao1 index provides an estimation of the community’s total species number, reflecting the overall abundance of biological entities [24]. Higher values of these indices signify greater species abundance. Additionally, the Shannon and Simpson indices are utilized to gauge the overall diversity within the communities of these samples. The Shannon index quantifies the total variety of taxa and their relative abundances, thereby illustrating the community’s complexity and evenness. Through the quantification of the species distribution’s uniformity and abundance, the Shannon index serves as an indicator of community diversity. A heightened index value signals a more equitable spread of species. On the other hand, the Simpson index evaluates the evenness and diversity of the species distribution within communities, with elevated values indicating a diminution in diversity [24]. Figure 2’s analysis on the Alpha diversity of mouse intestinal flora illustrates that the indices for Chao1, Shannon, Simpson, and observed_otus within the Model group surpass those in the Control group, implying that DSS treatment effectively enriches microbial diversity in mice. Following the administration of a low dose of polysaccharides (Low-DP), the aforementioned indices exhibit negligible deviation from those of the Model group, suggesting that a minimal polysaccharide intervention does not markedly influence the intestinal flora’s species richness or diversity in mice. Conversely, a high dose treatment of polysaccharides (High-DP) yields a reduction in these indices, aligning them more closely with the Control group’s levels. This change indicates an enhancement in bacterial diversity alongside a decrease in species richness, as a result of the High-DP treatment. 

### 3.2. The Effects of AAP on the β Diversity of Intestinal Flora in Mice

**Figure 3:**
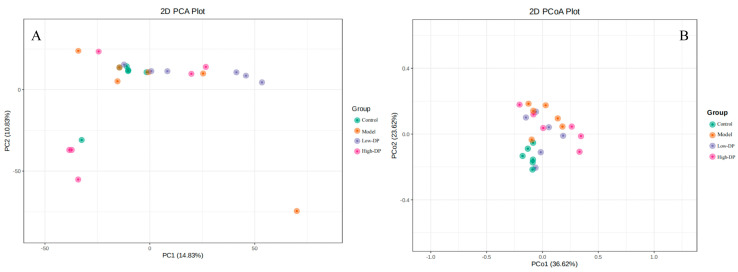
(**A**) Principal component analysis; (**B**) Principal coordinates analysis.

Beta diversity examines the variations in microbial community compositions across distinct samples by initially amalgamating OTU species annotations and abundance metrics from all samples into a comprehensive profiling table, effectively grouping identical classifications of OTUs [25]. This step is augmented by leveraging the phylogenetic relationships among OTUs to calculate the Unifrac distance, an unweighted measure that utilizes evolutionary linkages among microbial sequences within each sample to quantify inter-sample distances. When applied to multiple samples, this methodology generates a distance matrix based on these Unifrac distances, rooted in the abundance information of OTUs. Advanced statistical analyses, including principal component analysis (PCA) and principal coordinates analysis (PCoA), are then employed to discern the distinctions among various sample groups. The graphical outputs from PCA and PCoA illustrate a pronounced segregation between the Control group and the Model, Low-DP, and High-DP groups, with the latter three showing minimal separation amongst themselves (Figure 3). This indicates that while the administration of dandelion root polysaccharides might not significantly modify the gut microbiota structure in mice subjected to DSS-induced colitis, it appears to impact the microbiota composition in healthy mice, with a noted trend of clustering shifts in the High-DP group towards the Low-DP and Model groups, suggesting nuanced changes in the microbial community structure.

### 3.3. Effect of Polysaccharides from Dandelion Root on the Composition of Intestinal Flora in Mice

**Figure 4:**
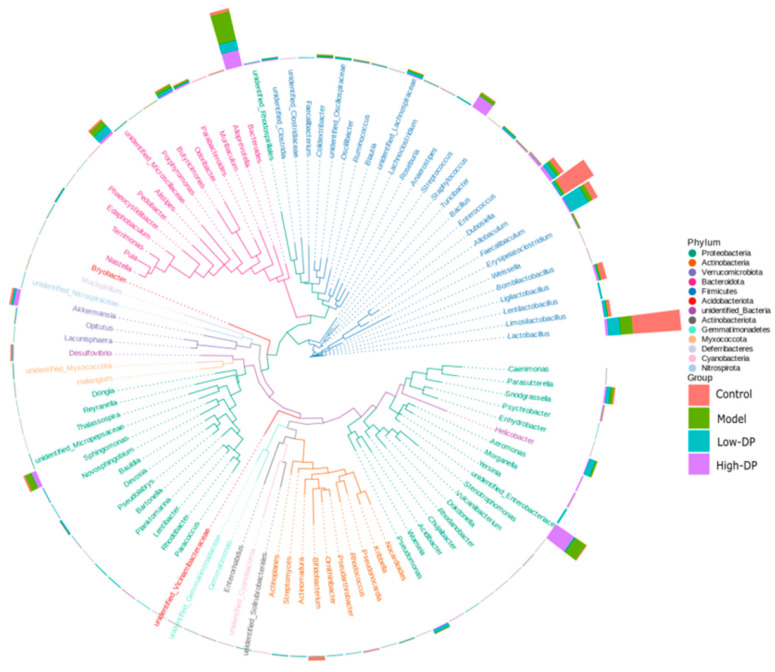
The top 100 abundance groups in a genus evolutionary tree information map.

To refrain from hastily concluding the lack of significant impact of dandelion root polysaccharide treatment on the gut microbiota of colitis-induced mice, a detailed analysis was conducted on the relative abundance of the intestinal flora at both phylum and genus levels, with the findings depicted in Figure 4. At the phylum level, among the predominant microbial phyla within the mouse gut microbiota, the twelve most abundant included Proteobacteria, Actinobacteriota, Verrucomicrobiota, Bacteroidetes, Firmicutes, Acidobacteriota (along with unidentified bacteria), Gemmatimonadetes, Myxococcota, Deferribacteres, Cyanobacteria, and Nitrospirata. Collectively, these phyla accounted for over 95% of the microbial composition in the samples, showcasing a marked stability in the microflora structure across the four groups at the phylum level. Given the constrained insights gleaned at the phylum level, a further examination was undertaken at the genus level to ascertain the composition of the mouse intestinal flora.

The analysis at the genus level revealed that the intestinal microbiota of mice primarily includes genera such as unidentified Enterobacteriaceae, Lactobacillus, Bacteroides, Alistipes, Escherichia coli, Allobaculum, Faecalibaculum, Dubosiella, Streptococcus, Helicobacter, Parasutterella, Ligilactobacillus, Limosilactobacillus, unidentified Lachnospiraceae, Sphingomonas, and Bifidobacterium, among others. Observations from the figure indicated that, in comparison to the Control group, genera such as Sphingomonas, Alistipes, Bacteroides, and Streptococcus, along with multiple instances of Helicobacter, showed an increase in relative abundance in both the Model group and the groups treated with Low/High doses of dandelion polysaccharide. Conversely, the prevalence of Helicobacter, Ligilactobacillus, Allobaculum, and Bifidobacterium experienced a decline. When comparing the Low-DP group with the Model group, there was an uptick in the relative abundance of Helicobacter, Parasutterella, Lactobacillus, Faecalibaculum, Dubosiella, and Alistipes, whereas the presence of Odoribacter and Sphingomonas diminished. The High-DP group, in contrast, exhibited an increased relative abundance of unidentified Enterobacteriaceae, Streptococcus, Bacteroides, and Akkermansia, while the abundance of Turicibacter decreased.

**Figure 5:**
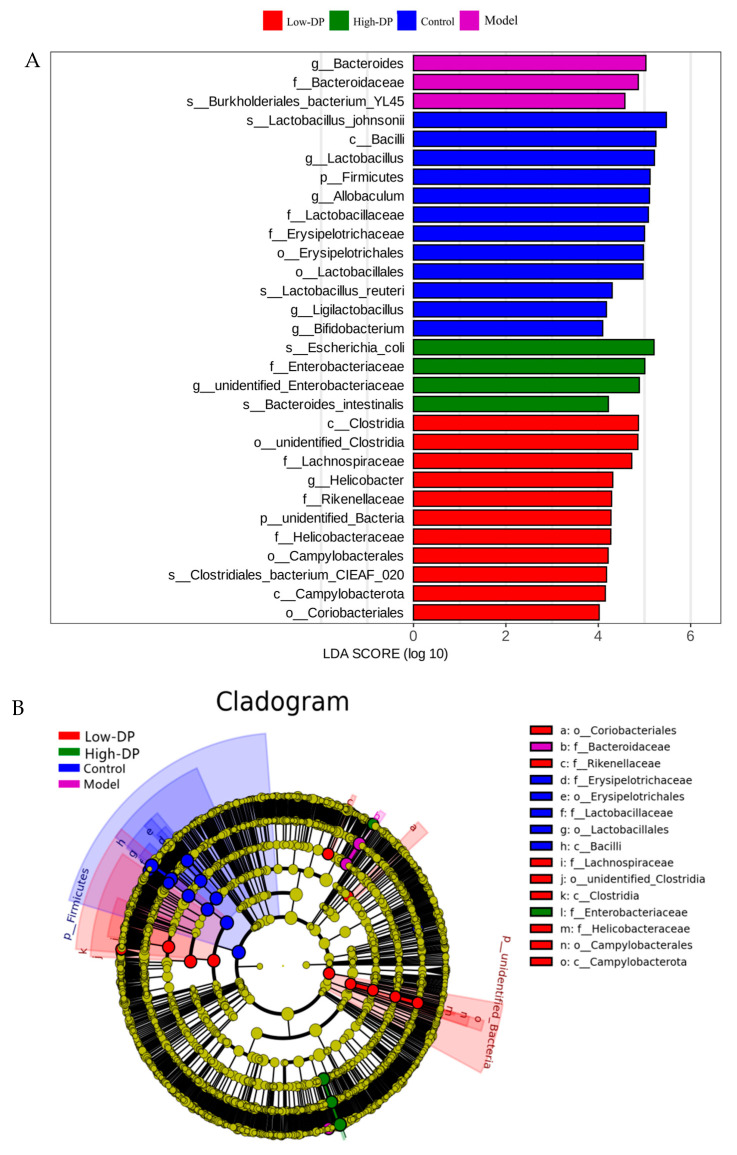

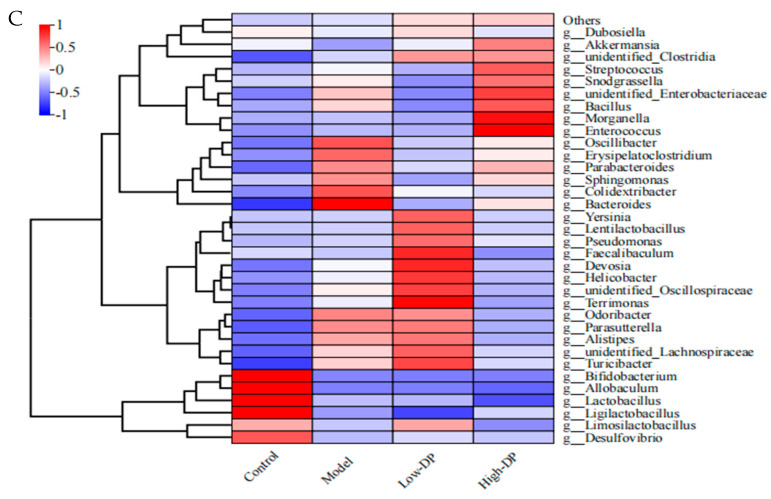
LEfSe analysis of mouse gut microbiota (*n* = 6). (**A**) LDA score histogram (LDA > 4); (**B**) Taxonomic clade diagram; (**C**) Heat map of different bacteria (*p* < 0.01).

To delve deeper into the influence of dandelion root polysaccharide on mycorrhizal associations, an analysis was conducted on species that exhibited significant differences in abundance across various taxonomic levels between the Control group and the Model, Low-DP, and High-DP groups using LEfSe. This analysis generated histograms of the linear discriminant analysis (LDA) scores (Figure 5A), highlighting species with notably different abundances across the groups. The threshold for significant difference was set at an LDA score of 4, with the histogram’s bar length indicating the magnitude of each species’ effect size. Notably, at the phylum level, significant variations were observed in the abundance of Firmicutes, unidentified bacteria, Actinobacteriota, Bacteroidota, and Proteobacteria, all key constituents of intestinal microbiota, marking substantial shifts in the cecal flora structure. At the genus level, the impact ranking of various bacteria in the Control, Model, Low-DP, and High-DP groups was determined to be Lactobacillus, Allobaculum, Bacteroides, unidentified Enterobacteriaceae, Helicobacter, Ligilactobacillus, and Bifidobacterium, signifying their respective influences.

The taxonomic branching diagram depicted in Figure 5B offers a detailed analysis of the differential bacterial abundance, highlighting the significant microbial phyla across various groups. The Firmicutes phylum emerges as a critical component within the gut microbiota of both the Control and Model groups, underscoring its integral role in preserving the equilibrium of intestinal microecology, fortifying the intestinal barrier, and orchestrating immune responses within the host. The steadfast presence of Firmicutes in the Control group contributes to the intestinal environment’s stability, suggesting a foundational role in health maintenance. Meanwhile, fluctuations in Firmicutes abundance within the Model group may correlate with disease dynamics, indicating its potential involvement in pathological processes. In the Low-DP group, the prominence of Firmicutes, coupled with Actinobacteria and unidentified bacteria, signifies their collective importance. Firmicutes are essential for the ongoing adaptative biological processes, whereas Actinobacteria play a pivotal role in decomposing complex carbohydrates and facilitating the synthesis of short-chain fatty acids, contributing to metabolic health [26]. The presence of unidentified bacteria hints at their participation in unique ecological roles, possibly involving competitive exclusion, establishing symbiotic relations, or engaging in distinct metabolic pathways, contributing to the gut’s overall health and resistance to pathogens. Within the High-DP group, the Ascomycota phylum’s notable role suggests a specialized function in bolstering defenses against microbial pathogens, highlighting the dynamic interplay between diet, microbiota, and host defense mechanisms.

Figure 5C highlights the changes in the genus-level relative abundance of key bacterial groups across different treatment groups. In the Control group, there was a notable increase (*p* < 0.05) in the abundance of beneficial genera such as Bifidobacterium, Allobaculum, and Lactobacillus. This suggests a healthy gut microbiota composition that potentially contributes to maintaining intestinal health. Conversely, the Model group experienced a significant rise in Bacteroides following DSS induction, implying that such an increase, along with a reduction in *Lactobacillus* spp., might be linked to more severe colonic inflammation due to DSS treatment. The Low-DP treatment led to a marked elevation in Faecalibaculum, indicative of a restorative effect on the gut’s mucosal barrier and a balancing act on the microbiota’s composition. This alteration suggests that increasing probiotics post-Low-DP treatment can suppress pathogenic bacteria, enhance both intestinal and systemic immune responses, and offer therapeutic benefits in mitigating UC symptoms and maintaining remission. In the High-DP group, there was a substantial rise in the relative abundance of Morganella and Enterococcus. This change is thought to result from a diminished presence of core beneficial bacteria, like those in the genus Bacteroides, which is paralleled by a notable increase in certain detrimental bacteria. Additionally, High-DP treatment might selectively inhibit or eliminate sensitive components of the intestinal flora, thereby providing a competitive advantage to resistant bacteria such as Morganella and Enterococcus, allowing these organisms more opportunities to thrive.

Within the intestinal ecosystem, Bacteroides, a genus within the Bacteroidetes phylum, stands out as a pivotal group capable of metabolizing a broad spectrum of dietary and host-derived polysaccharides. The fermentation of these polysaccharides by Bacteroides results in the production of short-chain fatty acids (SCFAs), notably butyrate, which plays a crucial role in maintaining gut health. Butyrate not only acts as an energy source for colonic epithelial cells but also supports the integrity of the intestinal barrier, mitigates inflammation, and potentially offers protective effects against inflammatory bowel diseases [27]. Lactobacillus, hailing from its namesake genus, is recognized as a foundational bacterial group and a beneficial component of the gut flora in both humans and animals. This genus collaborates with other anaerobic bacteria residing on the mucosal surface to create a biological shield. This barrier is essential for warding off external bacterial threats, stimulating immune functions, activating macrophages, and enhancing the host’s infection resistance and anti-tumor capabilities. Allobaculum, a genus proficient in carbohydrate metabolism, plays a pivotal role in generating butyric acid, a beneficial metabolic byproduct [25]. Polysaccharide interventions, notably with dandelion root polysaccharides, have been observed to enhance the concentration of n-butyric acid in fecal samples significantly. This increase in butyric acid concentration is instrumental in stabilizing the gut microbiota through the activation of the Nrf2 pathway [28]. It bolsters the intestinal barrier by promoting the expression of tight junction proteins, which, in turn, curtails the infiltration of deleterious substances and pathogens. The observed shifts in these key genera underscore the potential of SCFA metabolites, like butyric acid, to be synthesized via the fermentation of dandelion root polysaccharides [29]. This process indirectly activates the Nrf2 pathway, thereby fortifying intestinal antioxidant defenses, barrier integrity, and immune modulation. Consequently, this aids in preserving the micro-ecological balance within the gut, thereby fostering overall intestinal health [30]. These insights align with findings from prior research, affirming the beneficial impacts of polysaccharide-based interventions on gut microbiota dynamics.

### 3.4. Screening of Mouse Serum Biomarkers after DP Consumption

Utilizing non-targeted metabolomics approaches, this study embarked on an exploration of the comprehensive alterations in serum metabolites within mice subjected to DSS treatment, aiming to identify potential biomarkers. This investigative path paved the way for an in-depth analysis of the metabolic shifts occurring in mice with UC following DP intervention. Such an analysis endeavors to provide insight into the mechanisms through which Nrf2 modulation might influence UC progression by orchestrating changes in intestinal metabolism and microbial equilibrium. Consequently, this research seeks to shed light on the potential mechanisms through which DPs could contribute to the mitigation and alleviation of UC symptoms.

(1) UC biomarker identification and metabolic pathway analysis

**Figure 6:**
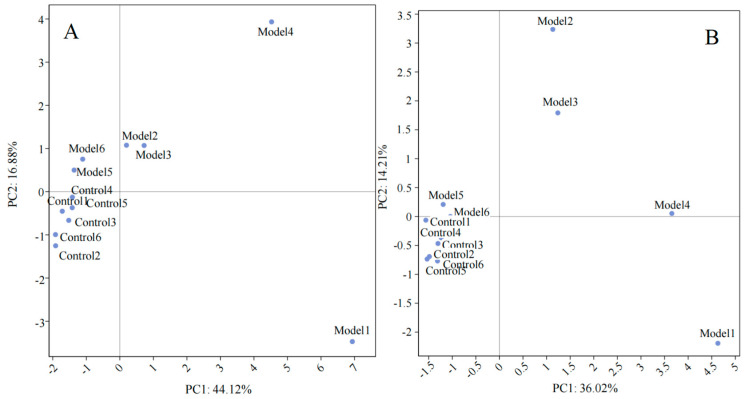
PCA of serum metabolites between Control group and Model group (*n* = 12. (**A**) Positive mode; (**B**) Negative mode.

Principal component analysis (PCA), an advanced technique in unsupervised multivariate statistics, is adept at evaluating the diversity among samples from various control and experimental setups. Employing this method, this study conducted a comprehensive examination of the differences in serum metabolites between mice in the Control group and those in the Model group, as illustrated in Figure 6. Through the analysis of data that had undergone normalization, a distinct separation was observable between the Control and Model groups in both positive and negative ion modes. This clear demarcation underscores the significant variation in the metabolic profiles between the two groups of mice, highlighting the profound impact of DSS treatment on the metabolic landscape of the mice.

**Figure 7:**
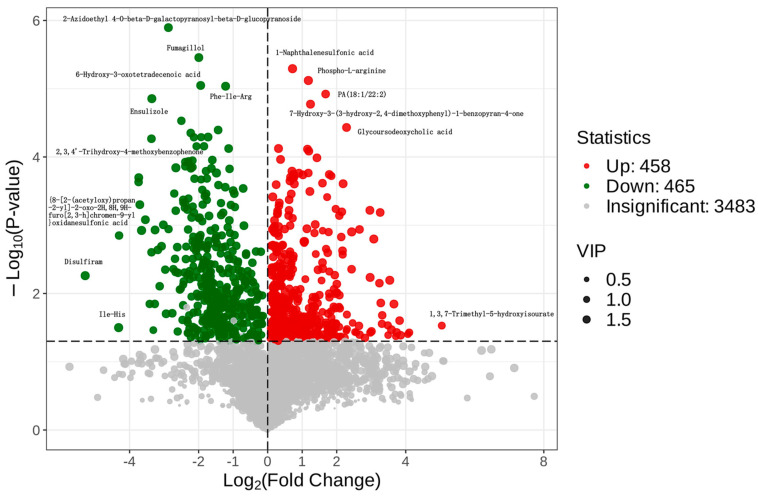
Volcano map of different metabolites in colon between Control group and Model group.

Through the application of orthogonal partial least squares discriminant analysis (OPLS-DA) on the metabolites identified in the Control and Model groups, a comprehensive search was conducted for differential metabolites linked to UC. This search employed a *t*-test analysis with specific criteria (Variable Importance in Projection (VIP) > 1, *p* < 0.01, and a fold change (FC) greater than 4 or less than 0.25) to pinpoint potential biomarkers. The analysis yielded a total of 3483 metabolites as potential biomarkers differentiating the Control from the Model group. The examination of these biomarkers through Volcano plot analysis (presented in Figure 7) revealed that 458 metabolites were significantly upregulated, while 465 metabolites were markedly downregulated. Notably, upregulated metabolites include 1-Naphthalenesulfonic acid, Phospho-L-arginine, PA(18:1/22:2), 7-Hydroxy-3-(3-hydroxy-2,4-dimethoxyphenyl)-1-benzopyran-4-one, and Glycoursodeoxycholic acid. In contrast, metabolites such as 2-Azidoethyl 4-O-beta-D-galactopyranosyl-beta-D-glucopyranoside, Fumagillol, (6R,8Z)-6-Hydroxy-3-oxotetradecenoic acid, Phe-Ile-Arg, Ensulizole, and 2,3,4’-Trihydroxy-4-methoxybenzophenone were significantly downregulated. These differential metabolites have the potential to serve as biomarkers for diagnosing and monitoring colitis.

**Figure 8:**
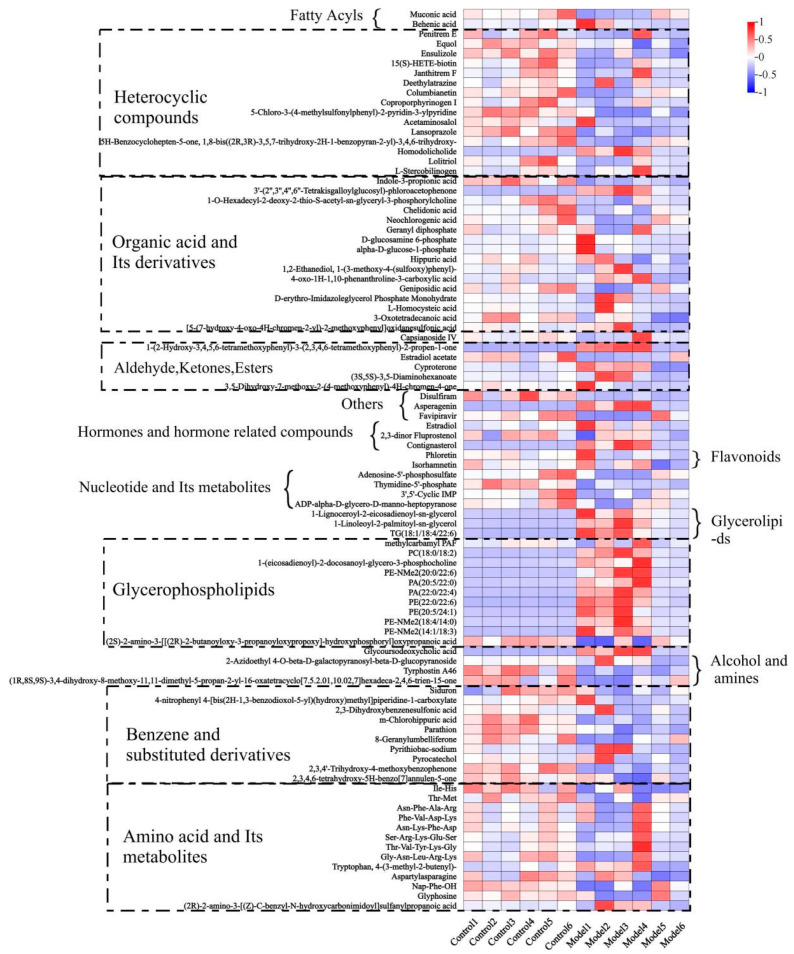
Control-Model heatmap of significant difference. Note: The number of upregulations and downregulations represent the number of upregulations and downregulations, respectively, of metabolite concentrations in the Model group compared to the Control group in the metabolite group to which they belong.

The enrichment analysis comparing the Control and Model groups, as depicted in Figure 8 through heatmaps, reveals significant variations in the concentrations of various metabolite groups. The Model group displayed considerably lower levels of metabolites such as fatty acyls, heterocyclic compounds, organic acids and derivatives, terpenoids, aldehyde and ketone esters, hormones and related substances, flavonoids, benzene derivatives, nucleotides, alcohols, amines, and amino acids. Conversely, metabolite groups like triglyceride lipids, glycerophospholipids, and bile acids exhibited notably higher concentrations compared to the Control group. 

**Figure 9:**
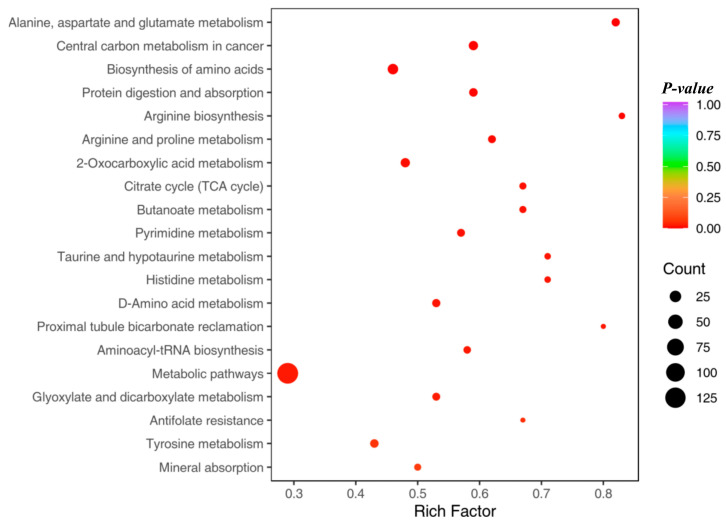
KEGG enrichment map of differential metabolites.

To enhance the understanding of metabolic function alterations in mice, a detailed examination was conducted on 3483 identified biomarkers through the Kyoto Encyclopedia of Genes and Genomes (KEGG) database. This in-depth analysis aimed to highlight specific KEGG metabolic pathways that were significantly influenced by the identified differential metabolites. Illustrated in Figure 9, the pathways are prioritized based on their *p* values, ascending from the most to the least significant. This prioritization revealed that a set of 151 differential genes between the Control and Model groups showed significant enrichment in six distinct KEGG pathways. These pathways predominantly pertain to the metabolism of amino acids within cells, the regulation of renal acid-base balance, and the metabolic processes involving taurine and hypotaurine, showcasing a complex interplay of metabolic functions impacted by the condition.

### 3.5. Changes in Serum Biomarkers in Mice after Dandelion Polysaccharide Intake

(1) Effects of DPs on key differential metabolites and metabolic pathway analysis

**Figure 10:**
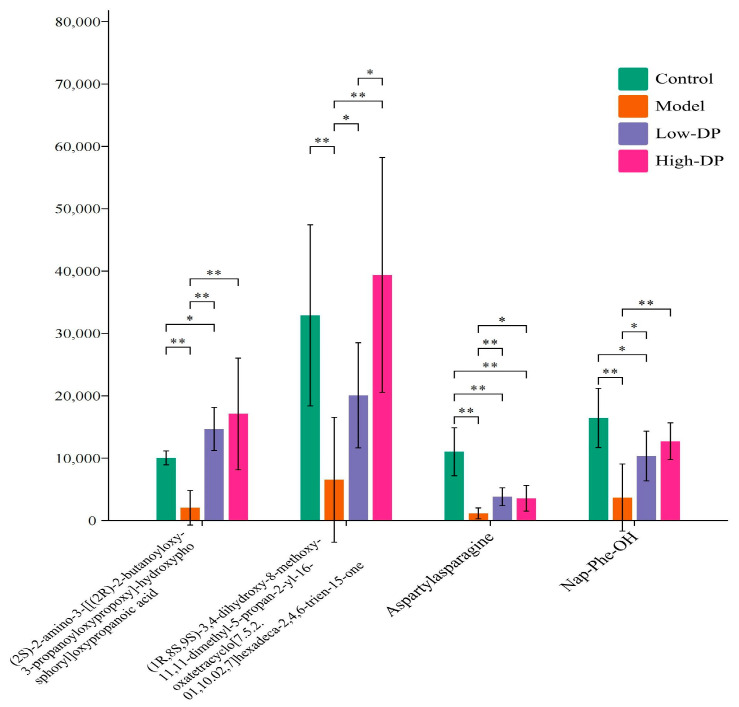
Trends of key differential metabolites in different groups. ”*” Indicates significance between the two groups (*p* < 0.05), ”**” Indicates highly significant difference between the two groups (*p* < 0.01).

Figure 10 highlights four critical differential metabolites identified across the Control, Model, Low-DP, and High-DP groups, namely (2S)-2-amino-3-[[(2R)-2-butanoyloxy-3-propanoyloxypropoxy]-hydroxyphosphoryl]oxypropanoic acid; (1R,8S,9S)-3,4-dihydroxy-8-methoxy-11,11-dimethyl-5-propan-2-yl-16-oxatetracyclo [7.5.2.01,10.02,7]hexadeca-2,4,6-trien-15-one; Aspartylasparagine; and Nap-Phe-OH. These metabolites exhibit unique structural characteristics and potential physiological roles: The first metabolite, which may be a derivative of phosphatidylserine, contains amino and carboxylic acid groups, along with a phosphate ester structure [31]. This composition suggests its involvement in maintaining the integrity of intestinal cell membranes and mitigating oxidative stress. It potentially does so by participating in the regulation of the gut’s antioxidant enzyme system, including superoxide dismutase (SOD), glutathione peroxidase (GPx), and catalase (CAT) [32]. The second and third metabolites, belonging to polycyclic ketones and containing hydroxyl, methoxy, and keto groups, may play a role in antioxidative processes. The hydroxyl group, in particular, is capable of reacting with free radicals, neutralizing them, and thereby halting chain reactions that contribute to oxidative stress, thus exhibiting antioxidant effects. By elucidating these metabolites’ structures and associated roles, this study sheds light on how DP interventions can influence the gut’s metabolic pathways, potentially reducing oxidative stress and supporting intestinal health by enhancing cellular energy metabolism, amino acid metabolism, the TCA cycle, and short-chain fatty acid production. These metabolic pathways are intricately linked to the gut’s antioxidant capabilities, suggesting that by modulating these pathways, DP intervention could improve the intestinal tract’s antioxidant defenses, thereby promoting overall gut health.

Aspartylasparagine, a dipeptide composed of aspartic acid and asparagine, plays a potentially beneficial role in the management and prevention of UC [33]. This is achieved through the modulation of intestinal epithelial cell activities, enhancement of intestinal barrier functions, and reduction of oxidative damage within the gut [34]. Furthermore, aspartic acid exerts a regulatory influence on bile acid metabolism within the enterohepatic system [35]. Research indicates that treatment with aspartic acid significantly stimulates the gene network responsible for bile acid synthesis and transportation in the enterohepatic axis. This action directly impacts the regulation of energy metabolism, encompassing lipid oxidation and bile acid metabolism, thereby contributing to overall digestive health and disease mitigation.

Nap-Phe-OH, a compound integrating naproxen (Nap) with phenylalanine (Phe), incorporates a hydroxyl (OH) group. Phenylalanine, an amino acid characterized by a benzene ring, possesses antioxidant capabilities due to the hydroxyl group attached to the ring [36]. Moreover, naproxen contributes to minimizing oxidative stress by its capacity to chelate metal ions, thereby reducing the formation of hydroxyl radicals through the decomposition of hydroperoxides. This dual functionality suggests a synergistic effect in combating oxidative damage, highlighting the potential therapeutic benefits of such compounds in oxidative stress-related conditions.

Figure 10 indicates that the content of the four key differential metabolites decreased in the Model group compared to the Control group, and the content of the four key differential metabolites was higher in the Low-DP and High-DP groups than in the Model group. This pattern suggests that the Model group might have undergone stress, leading to metabolic pathway adjustments in cells or organisms as a response to such stress. This situation could result in the heightened consumption of certain metabolites, thereby lowering their levels. The introduction of DPs to the Model group may have stimulated cellular defense mechanisms, notably the Nrf2 pathway, enhancing the antioxidant response. This intervention likely mitigated the stress-induced depletion of metabolites, culminating in a surge in their concentrations. This recovery not only indicates cellular adaptation but also repair mechanisms in response to stress. Consequently, it becomes essential to further explore the interplay between gut microbiota and these critical differential metabolites to uncover their interconnected roles.

**Figure 11:**
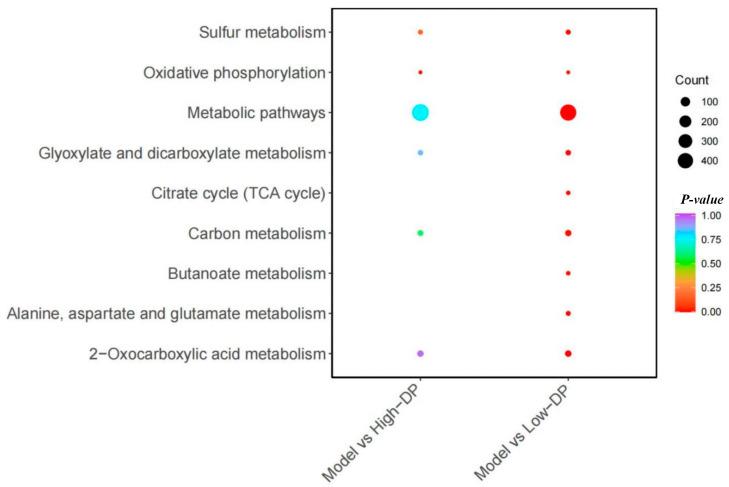
Model-Low-DP/High-DP KEGG pathway diagram of differential metabolites.

Figure 11 illustrates the distinct metabolic pathways active within the Model and Low-DP groups, emphasizing their involvement in several critical processes. These include the signaling and synthesis of various amino acids associated with the inflammatory response, cellular energy metabolism (primarily through oxidative phosphorylation), acetate and dicarboxylate metabolism (linked to amino acid metabolism), the tricarboxylic acid (TCA) cycle, carbon metabolism, butyrate metabolism, and the metabolism of alanine, aspartate, glutamate, and 2-oxocarboxylic acid. For the Model-High-DP group, the pathways predominantly focus on signal transduction related to the inflammatory response, the synthesis of diverse amino acids, and cellular energy metabolism (chiefly oxidative phosphorylation) [37]. The analysis reveals that the DP intervention impacts metabolic pathways majorly categorized under cellular energy metabolism (with an emphasis on oxidative phosphorylation), amino acid metabolism, the TCA cycle, and the production of short-chain fatty acids. All these pathways are intricately linked to the antioxidant capabilities of the intestinal tract. By modulating these pathways, DP intervention can significantly influence the intestinal tract’s antioxidant defenses, thereby positively affecting intestinal health. 

### 3.6. Correlation Analysis of Intestinal Flora and Metabolites in Mice after Intake of Auricularia Auricula Polysaccharides

**Figure 12:**
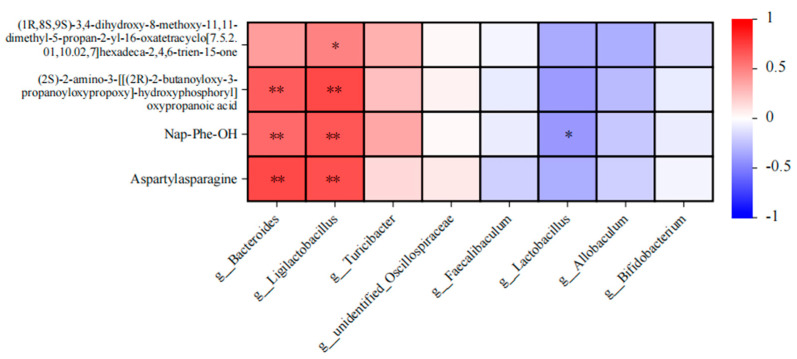
Heatmap of correlation between intestinal flora and metabolites. “*” Indicates significance between the two groups (*p* < 0.05), “**” Indicates highly significant difference between the two groups (*p* < 0.01).

In advancing the study of metabolic functions within the gut microbiota and the host, correlation analyses were undertaken to assess the relationship between predominant gut bacteria and crucial differential metabolites. Illustrated in Figure 12, Bacteroides demonstrated a significant positive correlation with four major differential metabolites. Similarly, Ligilactobacillus, a newly categorized genus of lactic acid bacteria, also showed a significant positive correlation with these key metabolites. These findings suggest that Bacteroides contributes to the production of short-chain fatty acids (SCFAs) by fermenting dietary fibers within the gut. Meanwhile, Lactobacillus genera, including Ligilactobacillus, are implicated in synthesizing antioxidants, such as Lactobacillin, to mitigate oxidative stress [38]. Collectively, these microorganisms produce antioxidant metabolites, including butyric acid and glutathione (GSH), which collaborate to counteract oxidative stress. They achieve this by directly scavenging free radicals, bolstering the intestinal barrier, modulating immune responses, and curbing inflammation, thereby safeguarding the intestine against oxidative harm and promoting gut health.

## 4. Conclusions

Through the analysis of gut flora, the relative abundance of Bifidobacterium, Allobaculum, and Lactobacillus significantly increased in the Control group (*p* < 0.05), the relative abundance of Bacteroides significantly increased in the Model group, and the relative abundance of Faecalibaculum significantly increased in the DP group in the DSS-induced mice. The relative abundance of Bacteroides in the Model group and Faecalibaculum in the DP group was significantly increased after DSS induction, indicating that polysaccharide intervention could significantly increase the content of n-butyric acid in the excretory fecal samples and that butyric acid could enhance the function of the intestinal barrier by activating the Nrf2 pathway, promote the expression of tight junction proteins, and reduce the infiltration of harmful substances and pathogens, thus contributing to the maintenance of the intestinal bacterial flora stability. There were four key metabolites that differed between the Control group, Model group, and DP group. Bacteroides had a significant positive correlation with the four key differential metabolites; Ligilactobacillus had a significant positive correlation with the four key differential metabolites, indicating that DP reduces oxidative stress through various mechanisms, such as direct scavenging of free radicals, enhancement of the intestinal barrier function, modulation of immune response, and inhibition of inflammation so as to protect the intestine from oxidative damage and maintain the health of the intestine.

## Data Availability

The datasets used or analyzed during the current study are available from the corresponding author upon reasonable request.
